# Gadolinium enhancement of cranial nerves: Implications for interstitial fluid drainage from brainstem into cranial nerves in humans

**DOI:** 10.1073/pnas.2106331118

**Published:** 2021-11-02

**Authors:** Aravinthan Varatharaj, Roxana O. Carare, Roy O. Weller, Mary Gawne-Cain, Ian Galea

**Affiliations:** ^a^Clinical Neurosciences, Clinical and Experimental Sciences, Faculty of Medicine, University of Southampton, Southampton SO17 1BJ, United Kingdom;; ^b^Wessex Neurological Centre, University Hospital Southampton National Health Service Foundation Trust, Southampton SO16 6YD, United Kingdom

**Keywords:** brain, interstitial fluid, drainage

## Abstract

Drainage of interstitial fluid and solutes from the brainstem has not been well studied. To map one drainage pathway in the human brainstem, we took advantage of the focal blood–brain barrier disruption occurring in a multiple sclerosis brainstem lesion, coupled with intravenous injection of gadolinium, which simulates an intraparenchymal injection of gadolinium tracer within the restricted confines of this small brain region. Using high-resolution MRI, we show how it is possible for interstitial fluid to drain into the adjacent trigeminal and oculomotor nerves, in keeping with a pathway of communication between the extracellular spaces of the brainstem and cranial nerve parenchyma.

Normally, interstitial fluid (ISF) and soluble metabolites are eliminated from brain tissue by diffusion through the extracellular spaces and then by rapid drainage along the walls of capillaries and arteries to lymph nodes, the intramural periarterial drainage (IPAD) pathway (1). Cerebrospinal fluid (CSF) drains into the arachnoid villi, meningeal lymphatics, and along channels adjacent to olfactory nerves, but there is little evidence for connections between these structures and the anatomical pathways for the drainage of ISF from the brain (2). The glymphatic hypothesis states that ISF drains along the walls of veins, but this is controversial and evidence from human pathological studies of cerebral amyloid angiopathy (occurring mainly in the walls of arteries and very rarely in veins) argues against the drainage of ISF along the walls of veins (3, 4).

Drainage of ISF and solutes from the brainstem has not been well studied. To map this drainage pathway in the human brainstem, we took advantage of the focal blood–brain barrier (BBB) disruption occurring in a multiple sclerosis (MS) brainstem lesion, coupled with intravenous injection of gadolinium, which simulates an intraparenchymal injection of gadolinium tracer within the restricted confines of this small brain region. We used high-resolution contrast-enhanced MRI to track the subsequent distribution of gadolinium in the adjacent cranial nerves.

## Results

**Fig. 1.**
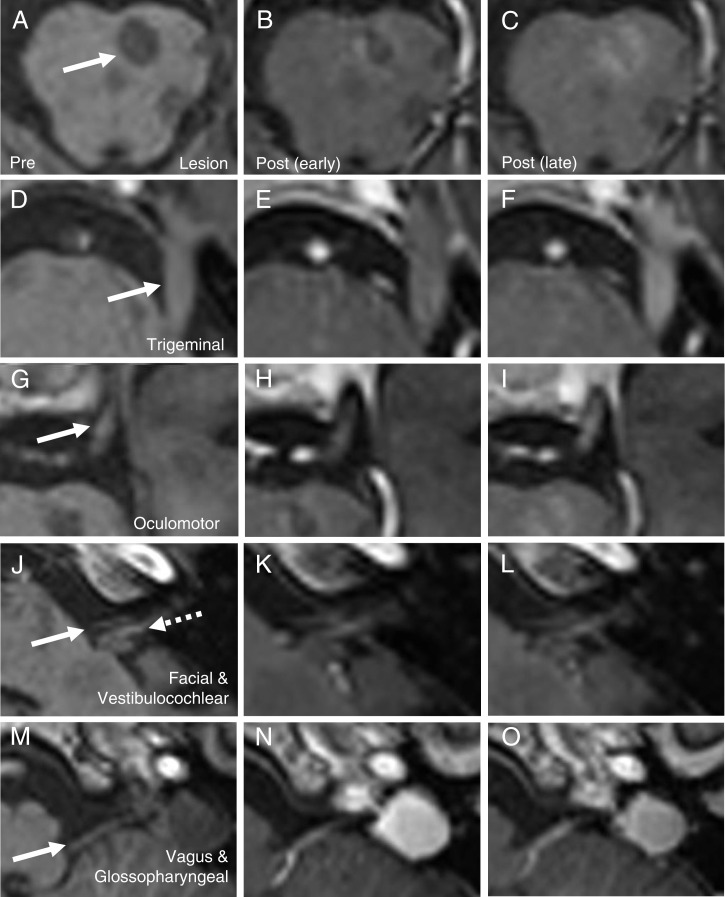
The evolution of contrast enhancement on T1-weighted images at early (7 min) and late (49 min) stages, for the pontine lesion (*A–C*), and ipsilateral trigeminal (*D–F*), oculomotor (*G–I*), facial and vestibulocochlear (*J–L*), and vagus and glossopharyngeal nerves (*M–O*). Arrows indicate the pontine lesion (*A*), trigeminal nerve (*D*), oculomotor nerve (*G*), facial nerve (*J*, solid arrow), vestibulocochlear nerve (*J*, dotted arrow), and the vagus/glossopharyngeal nerves (*M*).

**Fig. 2.**
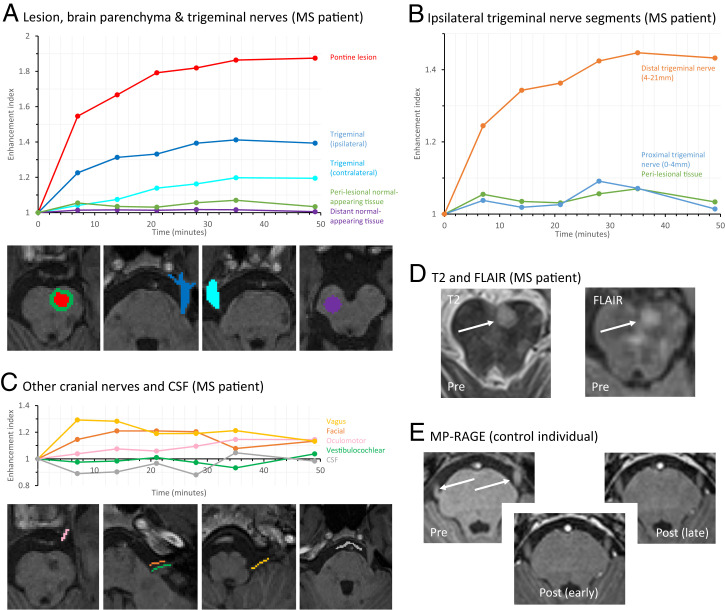
(*A*) Temporal profiles of enhancement for the pontine lesion, perilesional normal-appearing pontine tissue, distant pontine normal-appearing tissue, and both trigeminal nerves. The quantified ROIs are color-matched. (*B*) Temporal profiles of enhancement for the ipsilateral trigeminal nerve, divided into the proximal 4 mm (central segment) and the remainder of the distal nerve (peripheral segment). The profile for perilesional pontine tissue is overlaid for comparison. (*C*) Temporal profiles of enhancement and locations of ROIs for the ipsilateral oculomotor, facial, vestibulocochlear, vagus and glossopharyngeal nerves, and CSF in the prepontine cistern. Note that these nerves and therefore ROIs are significantly smaller than those in *A*, and consequently the temporal profiles are noisier. (*D*) Axial slices at the level of the superior pons with the lesion (arrows) visible on T2-weighted and FLAIR sequences. (*E*) Absence of contrast enhancement on T1-weighted images in the trigeminal nerves (arrows) of the control individual. This individual did not tolerate the full protocol and so the last time point is 28 min after contrast.

Images were acquired before intravenous injection of gadolinium-based contrast, and serially for 49 min in a 37-y-old man with a visibly enhancing pontine MS lesion, and a healthy 38-y-old man. The MS lesion was located in the left pons (Fig. 1*A*) and enhanced visibly after contrast (Fig. 1 *B* and *C*). The lesion was not associated with any appreciable swelling, the normal pontine contour was preserved, and the area of high T2-weighted signal (Fig. 2*D*) did not extend beyond the borders of the lesion, as indicated by T1-weighted hypointensity (Fig. 1*A*). Visible contrast enhancement was observed in the trigeminal (Fig. 1 *D*–*F*), oculomotor (Fig. 1 *G*–*I*), vagus and glossopharyngeal nerves (Fig. 1 *M*–*O*), but not in the facial and vestibulocochlear nerves (Fig. 1 *J*–*L*). The enhancement was greater on the ipsilateral side.

Quantitative analysis of signal intensity was performed, normalizing to precontrast signal. The pontine MS lesion showed rapid initial enhancement that progressively slowed, consistent with an exponential plateau pattern (Fig. 2*A*). Regions of interest (ROIs) were first placed over the trigeminal nerves, as they are of larger diameter compared to the other cranial nerves (Fig. 2*A*). The ipsilateral trigeminal nerve enhanced with a temporal profile similar to that of the MS lesion, but with reduced intensity (Fig. 2*A*). Contrast was present in the contralateral trigeminal nerve, although here the signal was dampened further (Fig. 2*A*). Contrast present in the perilesional normal-appearing tissue could be differentiated from normal-appearing pontine tissue distant from the lesion (Fig. 2*A*).

There were two possible sources for the trigeminal nerve enhancement: 1) contrast diffusing down the trigeminal nerve from the brainstem and 2) contrast derived from the blood supply to the nerve trunk. In order to distinguish between these two sources, we took advantage of the microanatomy of the trigeminal nerve. The first 4 mm of the nerve most proximal to the brainstem, referred to as the “central” portion of the nerve (5) (because it retains histological characteristics of CNS tissue), is devoid of vascularized perineurium and epineurium. On the other hand, the rest of the cranial nerve, referred to as the “peripheral” portion, has a perineurium and epineurium, which are vascularized and lack a blood–nerve barrier (6). Dividing the nerve into central and peripheral segments, the kinetics of enhancement were significantly different between the two (Fig. 2*B*), confirmed by a mixed ANOVA with repeated measures [*P* < 10^−6^, *F*(1, 201) = 27.4 for segment]. The timing of enhancement in the peripheral segment was earlier and larger in magnitude. On the other hand, the timing of the proximal segment mirrored that of the perilesional normal-appearing brain tissue, in keeping with continuity of ISF between the brainstem tissue and the trigeminal nerve endoneurium.

ROIs were placed over the oculomotor, facial, vestibulocochlear, and glossopharyngeal/vagus nerves. These nerves have a small diameter, approaching the limit of resolution of MRI, so partial volume effects are likely. Since no increase in signal was measured in the CSF (Fig. 2*C*), inclusion of surrounding CSF within the measured voxels would be expected to reduce the measured signal intensity. Despite this, there was still evidence of enhancement (Fig. 2*C*), with the exception of the vestibulocochlear nerve. It was not possible to accurately identify and quantitate the trochlear, abducens, accessory, and hypoglossal nerves, due to their small size or tortuousity and the resolution limit (1 mm^3^). No cranial nerve enhancement was observed in the control individual (Fig. 2*E*).

## Discussion

Lesion enhancement was consistent with BBB breakdown, delivering gadolinium contrast directly into the brainstem ISF, which then appeared within the cranial nerves. The timing and magnitude of the signal intensity within the central portion of the trigeminal nerve was similar to that of the normal-appearing tissue interposed between the lesion and the nerve, indicative of continuity of ISF spaces in the two compartments. The central portion of the trigeminal nerve is devoid of perineurium and endoneurium, which contributed to contrast enhancement in the distal nerve. Cranial nerves or their root entry zones were not inflamed, since no T2 signal abnormality was present in these areas.

Recent studies have suggested the presence of cells with lymphatic markers in cranial nerves (7, 8), but there are no lymphatic vessels in normal cranial or peripheral nerves (9) and drainage routes within nerves have yet to be clearly defined. With the presence of blood–nerve and perineurial barriers (10), it is possible that drainage in cranial nerves is similar to the brain (i.e., along the walls of capillaries and arteries) (1). Therefore, we propose that there is continuity between the basement membranes of capillaries in the brainstem (IPAD pathway) and trigeminal nerve endoneurium.

We were careful to select a MS lesion that was not visibly edematous, to minimize the likelihood that high local interstitial pressure opens up alternative pathways for ISF drainage. Nevertheless, the fact remains that we relied on MS pathology to deliver a high dose of gadolinium tracer into the brainstem parenchyma. Local inflammation would increase permeability of blood vessel walls. Structural changes to the IPAD pathway occur during MS, such as basement membrane damage secondary to matrix metalloproteinases (11), but whether ISF drainage is unchanged, increased, or decreased remains to be shown.

The drainage pathway of interstitial solutes from the brain along cranial nerves is of clinical importance. It may play a role in migraine, enabling calcitonin gene-related peptide (CGRP) released from the trigeminal nuclei within the brainstem to reach CGRP receptors in meningeal arteries to trigger vasodilatation (12). The same conduit may allow transport of intranasal therapeutics into the brainstem (13, 14). The pathway of communication between the extracellular spaces of the brain and nerve needs further study.

## Materials and Methods

The study protocol had institutional (University of Southampton Research Ethics Committee reference ERGO 46018) and National Health Research Authority (reference 18/LO/2015) approval. Written informed consent including publication of images was obtained after the participants had time to read information about the study and ask any questions, as per approved protocol. Full methods are in *SI Appendix*. In brief, imaging was performed on a 3T MR unit (Skyra, Siemens) using a 20‐element phased‐array head and neck coil. Three-dimensional magnetization prepared-rapid gradient echo (3D MP-RAGE) images covering the whole brain were acquired before contrast injection (Gd-DO3A-butrol; Bayer) (0 min), and at 7, 14, 21, 28, 35, and 49 min after injection, at a resolution of 1 mm^3^. Additional precontrast sequences included axial turbo-spin echo T2‐weighted and 3D FLAIR (fluid-attenuated inversion recovery). MP-RAGE images were affine-registered and read into MATLAB (Mathworks). Signal values at each time point were converted to enhancement indices by normalizing to precontrast signal. ROIs were drawn manually and the mean value within each ROI over time was calculated.

## Data Availability

Anonymized source images are available from the University of Southampton’s publicly accessible research repository at https://doi.org/10.5258/SOTON/D1824. All other study data are included in the article and *SI Appendix*.
